# Short-Term Impact of Low-Intensity Exercise with Blood Flow Restriction on Mild Knee Osteoarthritis in Older Adults: A Pilot Study

**DOI:** 10.3390/healthcare12030308

**Published:** 2024-01-25

**Authors:** Kang-Ho Kim, Seung-Ho Kang, Nackhwan Kim, Jaehyeong Choi, Seok Kang

**Affiliations:** 1 Department of Rehabilitation Medicine, Korea University Guro Hospital, Seoul 08308, Republic of Korea; kagho@naver.com (K.-H.K.); nackhwan@gmail.com (N.K.); 2 Department of Medical Device Industry, Dongguk University, Seoul 04620, Republic of Korea; rnd3@dsmaref.com; 3 Department of Rehabilitation Medicine, Armed Force Daejeon Hospital, Daejeon 34059, Republic of Korea; choijh810@gmail.com

**Keywords:** knee osteoarthritis, blood flow restriction, low-intensity exercise, immediate impact, physical performance, clinical symptom

## Abstract

This pilot study aimed to investigate the immediate impact of low-intensity exercises with blood flow restriction (BFR) on older adults with knee osteoarthritis (KOA). Fifteen patients with KOA who were over 50 years old, participated and underwent low-intensity resistance knee exercises at 30% of their one-repetition maximum with BFR three times/week for two weeks. Pre- and post-exercise assessments included pain levels, Western Ontario and McMaster Universities Osteoarthritis Index (WOMAC) scores, isokinetic knee strength, lower extremity muscle volume (via leg circumference and muscle thickness), functional performance tests (timed up-and-go [TUG] and sit-to-stand [STS]), skeletal muscle index (SMI) using bioelectrical impedance analysis, and handgrip strength (HGS). Post-exercise, there was a significant reduction in pain. WOMAC scores showed significant improvements across all three domains: pain, stiffness, and physical function. In the TUG and STS tests, completion times were significantly reduced. Thigh and calf circumferences, as well as thigh muscle thickness significantly increased after exercise. Post-exercise SMI and HGS also significantly increased. However, isokinetic knee strength did not show significant changes. In conclusion, low-intensity BFR exercises provide immediate benefits in symptoms and physical performance for patients with KOA, potentially inducing local and systemic muscle mass increase, even after a short-term intervention.

## 1. Introduction

Knee osteoarthritis (KOA) is a chronic degenerative disease that causes disability and pain [1]. KOA primarily involves the degeneration of cartilage and subchondral bone, accompanied by synovitis. Key factors exacerbating KOA include mechanical overload, inflammation, metabolic imbalances, hormonal changes, and ageing [2]. Common symptoms of KOA are pain, stiffness, limited joint mobility, and muscle weakness and these symptoms significantly impair daily activities and work performance, leading to a substantial economic impact on society [3,4].

Weakness in the quadriceps muscle is considered a significant risk factor for KOA [5] and is also a major determinant of physical function in patients with KOA [6]. Older patients with arthritis are at a higher risk of falls due to deficits in muscular strength and knee proprioception [7]. A reduction in muscle mass is associated with the presence and severity of age-related KOA [8]. Low muscle mass in the lower limb is independently associated with KOA [9]. Moreover, it is significantly related to knee pain in patients with KOA and is a useful clinical indicator for symptomatic KOA [10].

Exercise training is known to prevent cartilage degeneration, inhibit inflammation, and prevent loss of the subchondral bone and metaphyseal bone trabeculae [2]. A systemic review showed that the use of therapeutic exercise in patients with KOA can significantly reduce pain, and improve physical function and quality of life [11]. Strengthening exercises are highly recommended to restore muscle strength in patients with KOA. Strengthening training is beneficial for relieving pain, alleviating stiffness, enhancing muscle strength, improving physical function, and increasing the shock absorption ability of the lower extremity muscles during walking [12,13,14,15]. 

Conventionally, high-intensity resistance exercises (HIRE) with loads greater than 60% of one maximal repetition (1-RM) have been considered useful for gaining strength [16,17]. However, activities that place high loads on the knee affected by KOA can aggravate pain, swelling, and inflammation [18,19]. One challenge in encouraging long-term exercise for patients with KOA is the initial pain that can accompany exercise [20]. This pain can lead to reduced compliance with exercise regimens due to concerns that exercise is dangerous [21].

Blood flow restriction (BFR) has been known as a training method that can stimulate muscle growth without excessive resistance. BFR is achieved through applying a strap or a pneumatic cuff around the proximal region of the limbs. During exercises, arterial inflow is partially restricted and venous outflow is completely obstructed [22]. Typically, loads for BFR training are set at 20–30% of an individual’s 1-RM. Low-intensity resistance exercise (LIRE) with BFR, while utilizing lighter external loads compared to HIRE, induces reduced-to-similar adaptations in muscles [17,23], and comparable hypertrophy [17]. Although the exact mechanisms are still not well understood, the hypertrophic effects of resistance training with BFR may primarily result from an increased level of metabolic stress [24]. Furthermore, BFR training is reported to induce not only local but also systemic muscle growth by enhancing the levels of systemic hormones related to muscle growth [25,26].

LIRE with BFR can reduce joint stress during exercise, thereby enhancing tolerance to exercise in patients with KOA. It is also known that the pain level is lower during and after the exercise [27]. Previous studies have reported muscle hypertrophy post-BFR training in individuals with injuries [28,29]. Furthermore, BFR training is potentially effective in enhancing muscle strength in patients experiencing weakness and atrophy due to knee pathology and may serve as an effective clinical musculoskeletal rehabilitation tool [30,31].

Previous research results on the effects of BFR exercise for patients with KOA have shown varying outcomes. According to a study by Ferraz et al. [32], when HIRE, LIRE, and LIRE with BFR were performed over 12 weeks, the BFR group and the HIRE group showed similar significant effects on muscle strength and increased the muscle cross-sectional area compared to the LIRE group. In particular, BFR exercises were effective in reducing joint stress and improving pain, suggesting they could be a supplementary method for managing KOA. However, a study by Harper et al. [33], compared medium-intensity resistance exercise (MIRE) and LIRE with BFR over 12 weeks in patients aged over 60 years with KOA and demonstrated that the BFR exercise showed, on average, lower outcomes in terms of KOA symptoms, walking speed, and lower limb strength and function compared to MIRE.

A systemic review investigating the effect of BFR in patients who had undergone anterior cruciate ligament reconstruction or who were suffering from KOA demonstrated that LIRE with BFR could significantly increase the cross-sectional area of the quadriceps muscle, pain reduction, and improvement in quality of life, similar to that seen with HIRE [27]. However, another systematic review and meta-analysis by Granham et al. comparing BFR exercise with traditional resistance exercises in five studies on KOA found that the quality of evidence for all comparisons was low to moderate and showed no difference between BFR exercise and traditional resistance exercises [20]. That study concluded that the evidence favouring BFR exercise for patients with KOA is limited.

Evidence from previous studies suggests that BFR exercises for patients with KOA may be more effective in muscle strength improvement compared to low-intensity resistance exercises, and similar or slightly less effective than high or medium-intensity resistance exercises [20,23,27,32,33]. However, most studies on the impact of BFR exercises on patients with KOA were conducted throughout 4 to 12 weeks. To date, no study has explored the outcomes of BFR exercises conducted over periods shorter than two weeks. Our purpose was to explore the potential benefits and safety of a short-term BFR exercise program for individuals with KOA. Specifically, this pilot study aimed to determine whether a two-week program of BFR exercises alleviates the symptoms leads to functional improvement, localized and systemic muscle growth, and enhances strength in patients with KOA. 

## 2. Materials and Methods

### 2.1. Study Design and Participants

This was a prospective, single-group, pre-post design study. The study was approved by the Institutional Review Board of Korea University Guro Hospital (IRB No. 2021GR0556). We recruited adults aged ≥50 years who had been diagnosed with mild KOA (classified as Kellgren-Lawrence (KL) grade I or II), exhibited symptoms of KOA, and were not taking any medications for KOA or receiving other treatments (including physiotherapy). The exclusion criteria included individuals with any cardiovascular diseases, such as hypertension, heart failure, or ischemic heart disease, and those with a medical history of vasculitis or venous thrombosis/thromboembolism. All participants provided informed consent before they participated in the study and all procedures were conducted by the ethical standards of the 2008 Helsinki Declaration.

### 2.2. Intervention

Participants underwent LIRE at 30% of their 1-RM with a BFR program targeting the lower extremities, conducted three times/week for two weeks. The exercise program included leg presses, leg curls, and ankle pumps, performed using a chair and an elastic band (Thera-band; Hygienic Co., Akron, OH, USA). The 1-RM was estimated based on an equation considering the elastic force and length of each colour of the band [34,35]. The following prediction equation was then used: 1RM = resistance in kg/(1.0278 − [0.0278 × reps]) [36]. Each exercise was repeated 15 times per set, with each set lasting 1 min and incorporating 30 s of rest between sets, over a total of 5 sets. A digital pneumatic tourniquet (DTS-3000; Daesung Maref Co., Ltd., Gunpo-si, Gyeonggi-do, Republic of Korea) was applied for BFR to each proximal thigh region. During each set, the cuffs were inflated during the exercise phases and deflated during rest periods. The pressure level applied for BFR was set at 40% of the arterial occlusion pressure (AOP).

### 2.3. Assessments

We carried out evaluations to determine the impact and safety of a two-week BFR exercise program on patients with KOA, focusing on their clinical symptoms, physical status, and function. To confirm the effectiveness of the BFR exercise, we performed the test for changes in clinical symptoms, lower extremity functional status, muscle volume, isokinetic strength, and general muscle conditions before and after the 2-week program. For safety assessment, we conducted clinical blood tests before and after the program. Additionally, physical examinations were performed before and after each exercise session to promptly identify any adverse effects associated with the BFR exercise.

#### 2.3.1. Clinical Symptoms

Pain intensity was rated by the participants using a Numerical Rating Scale (NRS), an 11-point scale that ranges from 0 to 10, with 0 representing “no pain” and 10 representing “worst imaginable pain”. The Western Ontario and McMaster Universities Osteoarthritis Index (WOMAC) was used to assess health status related to KOA. The WOMAC evaluates three dimensions including pain, stiffness, and physical function. Each dimension is rated on a scale from 0 (no symptoms) to 4 (extreme symptoms), with higher scores indicating more severe impairment.

#### 2.3.2. Lower Extremity Function

To evaluate the changes in the functional status of patients with KOA following BFR exercise, we conducted the timed up-and-go (TUG) test and the sit-to-stand (STS) test (performed five times). In the TUG test, participants started seated in an armless chair. They were instructed to stand up, walk 3 m, turn around a marked tape, and return to the chair, doing so comfortably and safely [37]. In the STS test, participants were asked to stand up from the chair and sit back down five consecutive times as quickly as possible, without pausing. They were instructed to keep their arms folded across their chest throughout the test [38]. The duration of each test was measured with a stopwatch, accurate to the nearest 1/100 of a second. Both tests were conducted over two recorded trials, and the average time from these trials was used for data analysis. All the tests were performed under the supervision of one expert physical therapist.

#### 2.3.3. Muscle Volume

To assess muscle volume, we carried out anthropometric measurements of the lower limbs and ultrasonographic measurements of muscle thickness. The anthropometric measurements included the circumferences of both the thighs and calves. We used the same methods previously described in a study by Santos et al. [39]. For the thigh circumference measurements, participants were instructed to stand and shift their weight onto one leg while keeping the opposite knee slightly flexed and ensuring both feet stayed flat on the floor. We measured the circumference at the midpoint between the iliac crest and the knee of the non-weight-bearing leg. Calf circumference was measured at the point of maximum circumference while participants were seated, with the tape measure aligned perpendicular to the calf’s long axis. An inextensible steel measuring tape was used for all measurements, with special care taken to avoid compressing the subcutaneous tissue. All the measurements were carried out by two experienced physicians with over 10 years of practice. Before the study, the researchers had completed training in the measurement methods for a week to enhance inter-rater reliability, although they did not obtain the International Society for the advancement of kinanthropometry (ISAK) certification. To further minimize error, each participant was measured by the same researcher. Each measurement was taken three times at the same point, with the tape measure removed between each recording. We calculated the average of the three readings to ensure precision.

We measured the thickness of the rectus femoris (RF), vastus medial (VM), vastus lateralis (VL), and biceps femoris (BF) muscles using a portable ultrasound machine (HM70A; Samsung Medison Co., Ltd., Seoul, Republic of Korea) equipped with a linear array transducer (5–12 MHz). Participants were asked to lie in a supine on the examining table, with their legs extended and relaxed, for the measurement of the anterior thigh muscles. For the BF muscle measurement, participants were in the prone position. We placed the transducer at the most prominent muscle belly of each muscle as follows: for the RF muscle, at the midpoint between the anterior superior iliac spine (ASIS) and the superior pole of the patella; for the VM muscle, at the distal one-fourth point between the ASIS and medial patella; for the VL muscle, at the distal one-third point between the ASIS and lateral patella; and for the BF muscle, at the midpoint between the ischial tuberosity and the fibular head. The transducer was positioned perpendicular to the skin/musculature to minimize the risk of sampling a muscle obliquely. After identifying the muscle, the examiner slightly retracted the transducer to avoid compressing the muscle. Muscle thickness was measured using caliper-based tools included in the machine’s software. The process was repeated three times for each muscle, and the averaged values were recorded. All measurements were conducted by a single physician with over 10 years of experience.

#### 2.3.4. Isokinetic Knee Strength

The isokinetic strength of the knee flexors and extensors was measured using an isokinetic dynamometer (Humac NORM; CSMi Solutions, Stoughton, MA, USA). Peak torque was measured in concentric contraction at two velocities (60 and 180 deg/s) for knee flexors and extensors. The testing protocol involved three attempts at knee flexion and extension at these monitored velocities. Before testing at each velocity, participants completed four training trials at submaximal intensity.

#### 2.3.5. General Muscle Condition

In addition to evaluating the local physical status of the lower extremities, we assessed the general physical muscle status. This included the skeletal muscle index (SMI) using a bioelectrical impedance analysis (BIA) and general strength as indicated by handgrip strength (HGS). 

The BIA was performed using the InBody 720 device (InBody Co., Ltd., Seoul, Republic of Korea). As BIA is sensitive to hydration status, participants were instructed to avoid alcohol for 24 h and to fast for 2 h before the measurement. Additionally, the measurement was conducted at least 2 h post-exercise, under normal temperature conditions (20–25 °C). 

The measurement was carried out with participants in a standing posture. Before stepping onto the device platform, participants were instructed to wipe their feet and hands to improve conductivity. While standing upright on the platform equipped with embedded electrodes, they grasped the electrode handles, maintaining their arms straight and abducted at a 45-degree angle from the body. In total, eight electrodes were used, with two allocated to each foot and two to each hand. The electrode contact surfaces were cleaned with an alcohol swab before each measurement to reduce contact noise. The SMI was calculated by dividing the appendicular skeletal muscle mass by the square of the height [40,41]. 

HGS was measured using a hand-held dynamometer. Participants were seated comfortably with their feet flat on the floor and arms resting on a flat surface. The shoulder was maintained in a neutral position with the elbow flexion at a 90-degree angle and the wrist dorsiflexion at 0 to 30 degrees. Each participant held the dynamometer with the handle resting comfortably against the palm. They were instructed to squeeze the dynamometer with maximum effort, maintaining steady pressure for 3 to 5 s without sudden jerks or movements. The measurement was repeated three times and the highest value was recorded for each hand. 

#### 2.3.6. Safety

To prevent potential adverse events, we conducted physical examinations focused on the cardiovascular and musculoskeletal systems before and after each BFR exercise session. During the exercises, we monitored participants for any signs of discomfort or adverse responses, such as changes in heart rate, blood pressure, skin colour, and the presence of soft tissue edema. Clinical blood tests were performed to assess the inflammatory response, muscle damage, and increased risk of blood clots. These tests, including erythrocyte sedimentation rate (ESR), C-reactive protein (CRP), creatine phosphokinase (CPK), lactate dehydrogenase (LDH), myoglobin, lactic acid, and D-dimer, were conducted immediately before the initiation and after the completion of the two-week BFR exercise program.

### 2.4. Sample Size Determination and Statistical Analysis

The required sample size was calculated using G-power 3.1 software, based on a two-tailed test with an effect size of 0.8, an alpha of 0.05, and a power of 0.8. The resulting analysis indicated that a total of 15 participants would be necessary to achieve the desired statistical power for the Wilcoxon signed-rank test, which informed the recruitment target for the study.

Statistical analysis was performed using IBM SPSS 27.0 software. The Wilcoxon signed-rank test was applied to determine the difference between pre- and post-exercise values. Statistical significance was set at *p* < 0.05. Data analyzed were summarized as median values with interquartile ranges.

## 3. Results

### 3.1. Participants

Fifteen patients with mild KOA, aged ≥ 50 years, were enrolled in the study. The participants consisted of six males and nine females with a mean age of 59.20 years (standard deviation = 4.97). Of the participants, eight were classified as KL grade I, whereas the remaining seven were categorized as grade II (Table 1).

### 3.2. Clinical Symptoms

Knee pain assessed using NRS showed a statistically significant reduction after two weeks of the BFR exercise program. The WOMAC results also indicated a statistically significant decrease in symptoms following the exercise regimen. Significant improvements were observed in all subcategories including pain, stiffness, and physical function (Table 2).

### 3.3. Lower Extremity Function

**Figure 1:**
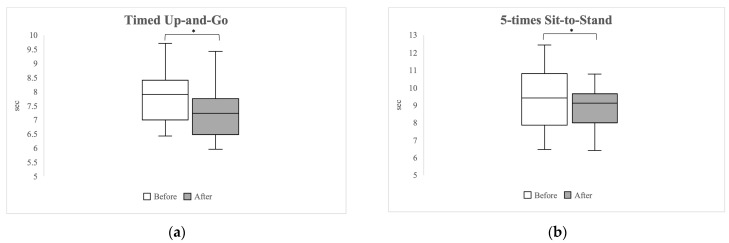
Boxplot graphs comparing results of the Timed Up-and-Go Test (**a**) and the 5-times Sit-to-Stand Test (**b**). Asterisks (*) denote statistically significant differences with *p*-values < 0.05.

Figure 1 shows the results of the TUG and STS tests. In the TUG test, the median time decreased significantly from 7.90 s (interquartile range [IQR] 7.00–8.40) pre-exercise to 7.23 s (IQR 6.48–7.75 s) post-exercise (*p =* 0.001). Similarly, in the STS test, a statistically significant change was observed with the time reducing from 9.41 s (IQR 7.85–10.81 s) pre-exercise to 9.11 s (IQR 7.98–9.65 s) post-exercise (*p =* 0.008).

### 3.4. Muscle Volume

#### 3.4.1. Anthropometric Measurements

**Figure 2:**
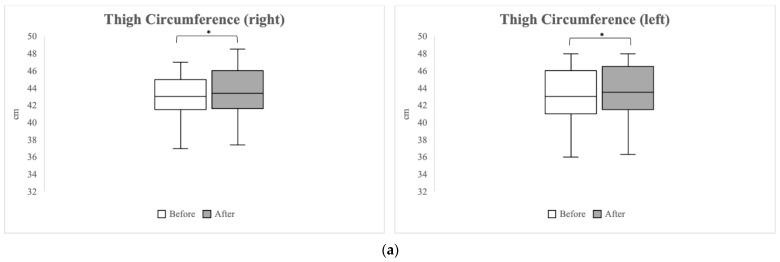

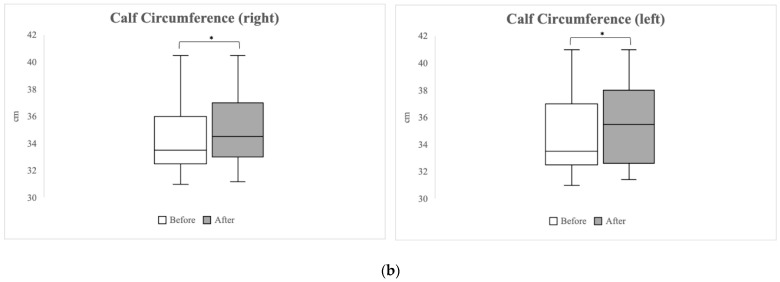
Changes in thigh (**a**) and calf circumference (**b**) after the 2-week blood flow restriction exercise program. Asterisks (*) indicate *p*-values < 0.05, denoting statistical significance.

A comparison of the lower extremity circumference pre- and post-exercise is presented in Figure 2. The right thigh showed a significant increase from 43.00 cm (IQR 41.50–45.00) pre-exercise to 43.40 cm (IQR 41.60–46.00 cm) post-exercise (*p =* 0.005). Similarly, the left thigh also exhibited a significant increase from 43.00 cm (IQR 41.00–46.00) pre-exercise to 43.50 cm (IQR 41.50–46.50 cm) post-exercise (*p =* 0.023). In the calf area, the right side showed a significant increase from 33.50 cm (IQR 32.50–36.00 cm) pre-exercise to 34.50 cm (IQR 33.00–37.00 cm) post-exercise (*p =* 0.033), and the left calf also demonstrated a significant increase, moving from 33.50 cm (IQR 32.50–37.00 cm) pre-exercise to 35.50 cm (IQR 32.60–38.00 cm) post-exercise (*p =* 0.003).

#### 3.4.2. Muscle Thickness

The muscle thickness changes were measured using ultrasonography. All the muscles measured on both sides showed a tendency to increase in thickness from pre-exercise to post-exercise after the two-week BFR exercise program. The left RF, bilateral VM, VL, and the left BF exhibited statistically significant changes (Table 3).

### 3.5. Isokinetic Knee Strength

No statistically significant changes in isokinetic knee strength were observed at a speed of 60 deg/s between the pre- and post-exercise. A statistically significant difference was noted in the left knee joint extension strength at a speed of 180 deg/s, but there was no significant difference in other measurements (Table 4).

### 3.6. General Muscle Condition

**Figure 3:**
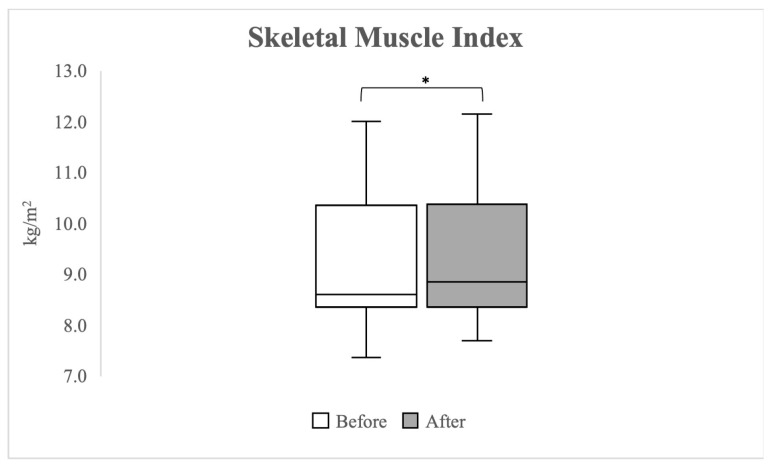
A boxplot graph comparing the skeletal muscle index, as measured by bioelectrical impedance analysis, before and after 2 weeks of the blood flow restriction exercise program. An asterisk (*) on the graph denotes values where *p <* 0.05, indicating statistical significance.

Figure 3 shows the changes in the SMI measured by BIA pre- and post-exercise. A significant change in SMI was observed from a median value of 8.61 kg/m^2^ (IQR 8.36–10.35 kg/m^2^) pre-exercise to 8.85 kg/m^2^ (IQR 8.35–10.38 kg/m^2^) post-exercise (*p* = 0.001).

**Figure 4:**
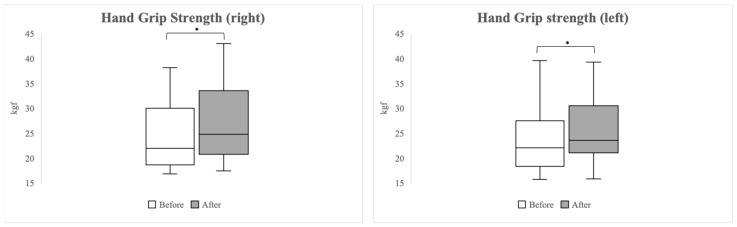
Changes in hand grip strength measured before and after the 2-week blood flow restriction exercise program. Asterisks (*) indicate *p*-values < 0.05, denoting statistical significance.

Figure 4 shows the comparison of HGS pre- and post-exercise. Following the BFR exercise program, there was a significant increase in HGS for both hands. On the right side, the median grip strength increased from 22.00 kgf (IQR 18.70–30.10 kgf) pre-exercise to 24.80 kgf (IQR 20.80–33.60 kgf) post-exercise (*p* = 0.003). On the left side, a significant increase was observed from a e median of 22.10 kgf (IQR 18.40–27.60 kgf) pre-exercise to 23.60 kgf (IQR 21.10–30.60 kgf) post-exercise (*p* = 0.029).

### 3.7. Safety

During or immediately after the exercise, there were no instances of skin colour change, swelling of the lower extremities, sensory abnormalities, muscle pain, or any signs of cardiovascular abnormalities observed in any of the participants. Clinical blood tests performed before the initiation of the BFR exercise program indicated that inflammatory markers, D-dimer levels, and muscle enzyme levels, including CPK, LDH, and myoglobin, were all within normal ranges for all participants. Follow-up tests after the two-week BFR exercise program showed no significant changes in ESR, CRP, d-dimer, CPK, LDH, or myoglobin levels. There was a statistically significant increase in lactic acid levels before and after the exercise program, but the levels remained within the normal ranges (Table 5).

## 4. Discussion

This pilot clinical study included 15 patients with mild KOA and aimed to evaluate the short-term effects and safety of LIRE with BFR, focusing on clinical symptoms, physical status, and functions. Despite the short duration and low intensity of the exercise program, there was a significant improvement in the clinical symptoms of KOA and the functionality of the lower extremities. Additionally, a notable increase in muscle volume of the lower extremity was observed, suggesting the immediate effectiveness of LIRE with BFR in promoting localized muscle growth. Furthermore, an increase in systemic muscle mass and general muscle function was also significant, even with the short-term exercise program.

In this study, a significant improvement in isokinetic knee strength was not observed. This could be due to the short duration (two weeks) of the exercise program, which might not have been sufficient for the muscle and neural adaptation process. A previous study on the time course of neuromuscular adaptation reported that rapid adaptation was observed after four weeks of exercise, eccentric, concentric and isometric strength occurred after this duration, while VL muscle hypertrophy could be seen as early as two weeks [42]. The results of our study showed a follow a similar pattern, suggesting that a longer duration might be necessary to achieve significant improvements in isokinetic knee strength. Nonetheless, our study results are meaningful and indicate a possibility of increased muscle volume and functional improvement even with such a short-term BFR exercise. 

Traditional HIRE is known to cause muscle fibre damage that leads to the release of cytokines, which activate the immune system and local satellite cells, resulting in phagocytosis of debris and tissue remodelling [43,44]. In contrast, BFR exercise is known to mediate muscle protein signalling and satellite cell proliferation through metabolic stress caused by BFR. This leads to increased recruitment of fast-twitch muscle fibres, inflammatory and endocrine responses, cellular swelling, and elevated intramuscular inorganic phosphates [44]. The intramuscular accumulation of metabolites due to BFR contributes to an increase in growth hormone levels and promotes the inflammatory response, thereby enhancing the production of myokines such as IL-6 [26]. The metabolic stress associated with glycogen depletion and hypoxia during BFR exercise is known to rapidly increase growth hormone and IL-6 levels without causing significant muscle damage, suggesting that it could be a potential mechanism by which BFR modulates acute inflammatory responses [44,45]. The clinical blood test results from this study showed no significant changes in muscle enzymes, but there was a significant increase in lactic acid levels post-exercise. This suggests that the effects of exercise might be related to metabolic stress without causing muscle damage. Thus, the immediate impact of the short-term BFR exercise is thought to result from rapid hormonal responses and cytokines that can modulate acute inflammatory responses. 

In this study, we evaluated not only the function, strength, and volume of local muscles under BFR but also assessed the impact of short-term BFR exercise on systemic muscle conditions and functions. SMI and HGS are useful indicators of systemic muscle condition and strength in older adults [46,47,48]. BFR exercise has been reported to enhance systemic muscle growth-related hormone levels and induce muscle growth in areas without BFR [25,26]. The results of this study confirmed that even short-term exercise can significantly increase SMI and HGS, indicating that BFR could be immediately beneficial in improving overall muscle condition.

This study found that short-term BFR exercise could help improve symptoms and function in KOA and also be effective in increasing overall muscle mass and strength. In a study by Song et al., knee pain levels in KOA had a strong negative relationship with moderate-intensity physical activities [49]. That study suggested that light activity may be a more acceptable way to increase physical activity than moderate activity. Traditional strengthening exercises, which commonly involve high resistance, may lead to joint pain in patients with KOA due to an increased load on the joints [33,49,50]. Patients with KOA might experience difficulty adhering to traditional HIRE, especially in the initial stages. As an alternative, LIRE combined with BFR may be more beneficial. This approach can be performed with reduced joint load and muscle damage. It has been shown to offer immediate symptom relief and functional improvements, even after a short-term program, as indicated in this study. Therefore, LIRE with BFR could be an effective method for managing KOA in an early stage. The positive effects observed in the initial stages over a short term can aid in maintaining ongoing exercise or in the transition to HIRE. The results of this pilot study could help in establishing such treatment protocols. Future comparative analyses between short-term HIRE and LIRE with BFR are needed to establish exercise treatment strategies for patients with KOA.

There are several limitations in this preliminary pilot study. The primary limitation is the study design, specifically the use of a one-group pre-test and post-test design without a control group. Because this study was a preliminary exploratory clinical trial for a future randomized case-control study of the effects of a short-term BFR exercise program in patients with mild KOA, a control group was not recruited. This decision, made in the context of the study’s initial design as a pilot investigation, significantly impacts the study’s internal validity. The absence of a control group and the pre-post design of the study preclude the establishment of causality. This limitation is acknowledged, and the results should be interpreted with caution, considering this design constraint. Furthermore, we performed an additional analysis using ANCOVA, in which age, sex, and BMI were incorporated as covariates. This analysis, included in the Supplementary Material, aims to mitigate the influence of these potential confounding factors and to provide a more nuanced understanding of our primary outcome variables.

The second limitation is the small sample size of participants (*n* = 15). While this number met the initial sample size calculations, it restricts the statistical power and generalizability of the results. This limitation is particularly relevant given the use of nonparametric tests, which may not fully represent the dynamics of a small sample.

Thirdly, the study’s focus on patients with mild knee osteoarthritis (KOA) limits the applicability of the findings to those with more severe conditions. This specificity in participant selection suggests that the results may not be representative of the broader KOA patient population.

Finally, the lack of a sequential time-order analysis in the study design did not allow for the confirmation of the immediate response and the duration of the effects of the BFR exercise. This is a significant limitation as it hinders our ability to assess the short-term and long-term impacts of the intervention, which are critical for understanding the full scope of its efficacy.

In response to these limitations, future research will involve a randomized case-control design with a larger sample size. This approach aims to provide a more comprehensive understanding of the effects of BFR exercise and address the limitations of the current pilot study. By incorporating a control group and expanding the participant profile, we intend to offer more conclusive insights into the effectiveness of BFR exercise across varying severities of KOA.

## 5. Conclusions

This pilot study has confirmed that short-term LIRE with BFR has an immediate positive effect on lower extremity muscle strength, function, and symptoms of KOA in older patients. Furthermore, BFR exercise is effective in not only promoting localized muscle growth but also increasing systemic muscle volume and strength even in a short period. It is expected to improve local and systemic muscle function. Therefore, BFR exercise could be considered as an initial management method for older patients with KOA.

## Figures and Tables

**Table 1:** Demographic data of the participants.

Gender (male%)	40%
Age (years)	59.20 ± 4.97
Height (cm)	159.80 ± 6.30
Weight (kg)	64.05 ± 9.70
BMI	24.99 ± 2.63
KL grade I (n)	8
KL graded II (n)	7

BMI (Body Mass Index), KL grade (Kellgren-Lawrence grade). Values of age, height, weight, and BMI are presented as the mean ± standard deviation.

**Table 2:** Changes in clinical symptoms following the blood flow restriction exercise program.

	Pre-Exercise	Post-Exercise	*p*-Value
Knee pain (NRS)	2.00 (1.00–4.00)	1.00 (0.00–2.00)	0.004
WOMAC-Pain	2.00 (1.00–5.00)	1.00 (0.00–3.00)	0.013
WOMAC-Stiffness	1.00 (0.00–3.00)	0.00 (0.00–2.00)	0.039
WOMAC-Physical function	4.00 (2.00–8.00)	2.00 (0.00–10.00)	0.008
WOMAC-Total	7.00 (3.00–17.00)	3.00 (0.00–16.00)	0.003

NRS (Numeric Rating Scale), WOMAC (Western Ontario and McMaster Universities Osteoarthritis Index). All values are presented as the median (interquartile range). *p*-values are calculated by the Wilcoxon signed-rank test.

**Table 3:** Changes in thigh muscle thickness following the blood flow restriction exercise program.

Muscles	Pre-Exercise (cm)	Post-Exercise (cm)	*p*-Value
Right RF	1.81 (1.57–1.97)	1.87 (1.69–2.09)	0.061
Left RF	1.82 (1.42–2.29)	1.92 (1.54–2.15)	0.041
Right VM	2.95 (2.65–3.62)	3.19 (2.72–3.94)	0.015
Left VM	2.86 (2.53–3.89)	3.19 (2.69–4.15)	0.011
Right VL	2.22 (1.86–2.60)	2.51 (2.30–2.84)	0.002
Left VL	2.20 (1.70–2.50)	2.50 (2.24–2.81)	0.014
Right BF	2.88 (2.53–3.33)	2.98 (2.63–3.53)	0.088
Left BF	2.68 (2.49–2.94)	3.28 (2.81–3.41)	0.001

RF (Rectus femurs), VM (Vastus medialis), VL (Vastus lateralis), BF (Biceps femoris). All values are presented as the median (interquartile range). *p*-values are calculated by the Wilcoxon signed-rank test.

**Table 4:** Comparison of isokinetic knee strength before and after the blood flow restriction exercise program.

		Pre-Exercise (Nm)	Post-Exercise (Nm)	*p*-Value
60°/s	Right KE	75.00 (64.00–113.00)	77.00 (62.00–96.00)	0.887
	Left KE	81.00 (66.00–123.00)	79.00 (71.00–107.00)	0.319
	Right KF	37.00 (24.00–58.00)	39.00 (34.00–56.00)	0.073
	Left KF	39.00 (27.00–66.00)	45.00 (31.00–58.00)	0.379
180°/s	Right KE	48.00 (41.00–73.00)	52.00 (39.00–72.00)	0.197
	Left KE	50.00 (41.00–77.00)	50.00 (45.00–85.00)	0.018
	Right KF	24.00 (18.00–39.00)	27.00 (20.00–45.00)	0.148
	Left KF	24.00 (18.00–41.00)	27.00 (20.00–37.00)	0.674

KE (knee extensor), KF (knee flexor). All values are presented as the median (interquartile range). *p*-values are calculated by the Wilcoxon signed-rank test.

**Table 5:** Comparison of isokinetic knee strength pre- and post-blood flow restriction exercise program.

	Pre-Exercise	Post-Exercise	*p*-Value
CRP (mg/L)	0.66 (0.17–0.86)	0.47 (0.32–1.07)	0.530
ESR (mm/h)	16.00 (5.00–21.00)	16.00 (4.00–21.00)	0.752
D-dimer (μg/mL)	0.27 (0.27–0.32)	0.27 (0.27–0.34)	0.088
CPK (IU/L)	87.00 (65.00–160.00)	89.00 (76.00–161.00)	0.139
LDH (IU/L)	360.00 (311.00–402.00)	363.00 (284.00–451.00)	0.955
Myoglobin (ng/mL)	27.00 (21.00–30.40)	27.60 (21.00–31.20)	0.878
Lactic acid (mmol/L)	1.50 (1.30–1.90)	2.20 (1.30–2.70)	0.047

All values are presented as the median (interquartile range). *p*-values are calculated by the Wilcoxon signed-rank test.

## Data Availability

The data presented in this study are available on request from the corresponding author. The data are not publicly available due to privacy restrictions.

## Supplementary Materials

The following supporting information can be downloaded at: https://www.mdpi.com/article/10.3390/healthcare12030308/s1, Table S1: Results of ANCOVA.
