# Supplementary material for: Short-Term Impact of Low-Intensity Exercise with Blood Flow Restriction on Mild Knee Osteoarthritis in Older Adults: A Pilot Study
Source: Healthcare (Basel). 2024 Jan 25;12(3):308. doi: 10.3390/healthcare12030308 (PMC10855245; doi:10.3390/healthcare12030308)
Supplement: Supplementary file 1 [file healthcare-12-00308-s001.zip › healthcare-2791718-supplementary.pdf]

	Timed up-and-go			Sit-to-stand			Skeletal muscle index			Hand grip strength (Right)			Hand grip strength (Left)		
	F	p-value	parital η2	F	p-value	parital η2	F	p-value	parital η2	F	p-value	parital η2	F	p-value	parital η2
Adjusted model	5.177	0.016	0.742	3.279	0.001	0.863	328.380	0.000	0.995	38.608	0.000	0.955	37.001	0.000	0.954
Pre-exercise	22.746	0.001	0.717	26.584	0.001	0.747	407.768	0.000	0.978	86.684	0.000	0.906	77.112	0.000	0.895
Gender	1.587	0.239	0.150	0.759	0.406	0.078	0.047	0.833	0.005	0.015	0.904	0.002	0.110	0.748	0.012
Age	0.913	0.364	0.092	2.305	0.163	0.204	2.853	0.125	0.241	0.377	0.554	0.040	2.934	0.121	0.246
BMI	0.174	0.686	0.019	7.622	0.022	0.459	0.294	0.601	0.032	1.015	0.340	0.101	12.310	0.007	0.578
KL-grade	0.067	0.802	0.007	0.235	0.639	0.025	1.902	0.201	0.174	0.233	0.641	0.025	0.015	0.906	0.002

	Thigh circumference (Right)			Thigh circumference (Left)			Calf circumferemce (Right)			Calf circumferemce (Left)		
	F	p-value	parital η2	F	p-value	parital η2	F	p-value	parital η2	F	p-value	parital η2
Adjusted model	76.868	0.000	0.977	81.902	0.000	0.798	25.520	0.000	0.934	48.440	0.000	0.964
Pre-exercise	209.995	0.000	0.959	201.785	0.000	0.951	3.970	0.077	0.306	34.063	0.000	0.791
Gender	2.917	0.122	0.245	2.032	0.188	0.184	5.212	0.048	0.367	0.102	0.757	0.011
Age	1.453	0.259	0.139	0.218	0.652	0.024	8.080	0.019	0.473	0.385	0.550	0.041
BMI	0.058	0.815	0.006	0.000	0.994	0.000	27.954	0.001	0.756	0.139	0.718	0.015
KL-grade	1.479	0.255	0.141	0.005	0.946	0.001	6.200	0.034	0.408	0.245	0.633	0.026
